# Supplementary material for: Initial Validation for the Estimation of Resting-State fMRI Effective Connectivity by a Generalization of the Correlation Approach
Source: Front Neurosci. 2017 May 16;11:271. doi: 10.3389/fnins.2017.00271 (PMC5433247; doi:10.3389/fnins.2017.00271)
Supplement: Supplementary file 1 [file Presentation1.PDF]

Supplementary Material: Initial Validation for the Estimation of Resting-state fMRI Effective Connectivity by a Generalization of the Correlation Approach

Nan Xu*, R. Nathan Spreng and Peter C. Doerschuk

*Correspondence:
Nan Xu
nx25@cornell.edu

$$\mathrm{BIC} = N_x \log\left(\frac{2\pi}{N_x - N_{h_{j|i}}}\mathcal{J}(h_{j|i})\right) + N_x - N_{h_{j|i}} + N_{h_{j|i}} \log(N_x) \quad \text{(S1)}$$

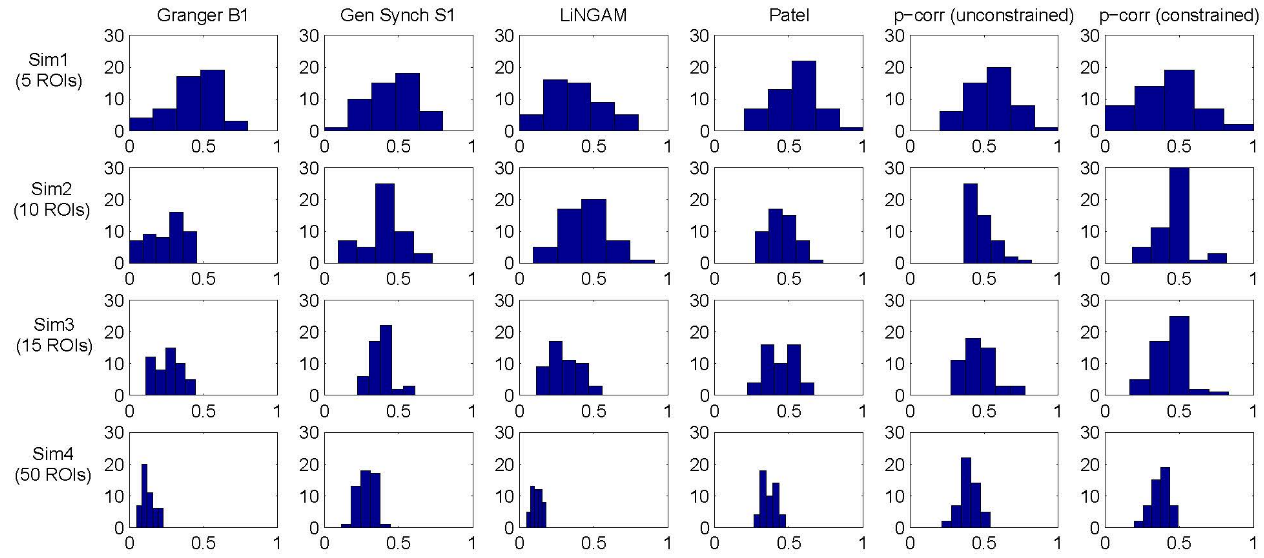

Figure S1. Accuracy histogram for Granger B1, Gen Synch S1, LiNGAM, Patel and p-correlation with unconstrained and constrained Least Squares.

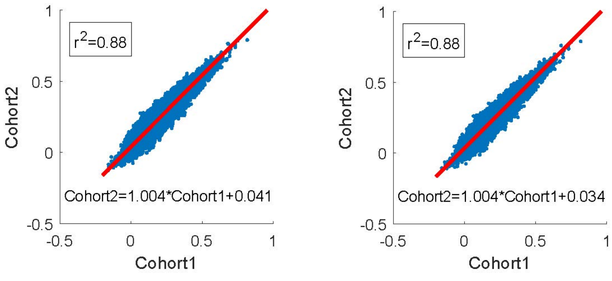

(a) p-correlation (b) correlation

Figure S2. Scatter plot of p-correlation and correlation for the two cohorts. The red line is the Least Squares fit for predicting Cohort 2 from Cohort 1.
